# What makes a good life: using theatrical performance to enhance communication about polygenic risk scores research in patient and public involvement

**DOI:** 10.1007/s12687-023-00635-1

**Published:** 2023-02-10

**Authors:** Amy M. Mason, Ifunanya Obi, Olamide Ayodele, Samuel A. Lambert, Sarah Fahle

**Affiliations:** 1 British Heart Foundation Cardiovascular Epidemiology Unit, Department of Public Health and Primary Care, University of Cambridge, Cambridge, UK; 2 Medical Research Council Biostatistics Unit, Cambridge Institute of Public Health, University of Cambridge, Cambridge, UK; 3 Heart and Lung Research Institute, University of Cambridge, Cambridge, UK; 4 London, UK; 5 Cambridge Baker Systems Genomics Initiative, Department of Public Health and Primary Care, University of Cambridge, Cambridge, UK; 6 Health Data Research UK Cambridge, Wellcome Genome Campus and University of Cambridge, Cambridge, UK; 7 National Institute for Health and Care Research Blood and Transplant Research Unit in Donor Health and Behaviour, University of Cambridge, Cambridge, UK

## Abstract

The aim of this patient and public involvement and engagement (PPIE) work was to explore improvised theatre as a tool for facilitating bi-directional dialogue between researchers and patients/members of the public on the topic of polygenic risk scores (PRS) use within primary or secondary care. PRS are a tool to quantify genetic risk for a heritable disease or trait and may be used to predict future health outcomes. In the United Kingdom (UK), they are often cited as a next-in-line public health tool to be implemented, and their use in consumer genetic testing as well as patient-facing settings is increasing. Despite their potential clinical utility, broader themes about how they might influence an individual’s perception of disease risk and decision-making are an active area of research; however, this has mostly been in the setting of return of results to patients. We worked with a youth theatre group and patients involved in a PPIE group to develop two short plays about public perceptions of genetic risk information that could be captured by PRS. These plays were shared in a workshop with patients/members of the public to facilitate discussions about PRS and their perceived benefits, concerns and emotional reactions. Discussions with both performers and patients/public raised three key questions: (1) can the data be trusted?; (2) does knowing genetic risk actually help the patient?; and (3) what makes a life worthwhile? Creating and watching fictional narratives helped all participants explore the potential use of PRS in a clinical setting, informing future research considerations and improving communication between the researchers and lay members of the PPIE group.

**Supplementary Information:**

The online version contains supplementary material available at 10.1007/s12687-023-00635-1.

## Introduction

Patient and public involvement and engagement (PPIE), which enhances the quality and appropriateness of research, is a core component of healthcare research in the United Kingdom (UK). “Involvement” is research carried out “with” or “by” members of the public rather than “to”, “about” or “for” them (National Institute for Health and Care Research 2021). “Engagement” is where information and knowledge about research are provided and disseminated (National Institute for Health and Care Research 2021). In order for healthcare to be equitable, it is essential that data, participation in clinical trials and participation in research are representative of the population. Yet, the majority of the public is rarely exposed to healthcare research directly (Höttecke and Allchin 2020) and thus it can be difficult for researchers to explain, and engage in meaningful dialogue, about their work. Researchers are also trained to use highly specific terminology and present their findings in formal ways, such as academic papers and conferences, which can be unhelpful and uninviting to the public.

Theatre communication is the antithesis of such carefully documented academic communication (Vanover 2021) and may avoid some of the known drawbacks in traditional presentation styles (Wecker 2012; Baker et al. 2018). One advantage is in the sustained emotional impact, both for the researcher and the audience (Hundt et al. 2019; Gray et al. 2000). There is a rich history of using theatre to collect, disseminate and validate health research, engaging both the creator and the audience more concretely with the research findings (Stuttaford et al. 2006; Rossiter et al. 2008; Kerr et al. 2020). Rossiter et al. (2008) report how theatrical research-based performance can use the creative power of theatre as an interpretive, analytic tool. Furthermore, they argue that theatrical research-based performance allows greater space for discussion by removing the restrictions imposed by communicating solely about facts. The paper also notes the lack of strong evidence of the efficacy of using theatre and attributes this to the inherent difficulty of evaluating artistic intervention. However, it is well documented that fictional narratives in media other than theatre can impact public health activity, with televised soap operas dramatically increasing health screening (Howe et al. 2002; Richardson et al. 2002), and future health choices (Mejia et al. 2017; Vaughan et al. 2000). Henderson and Kitzinger (1999) report that dramatized personal accounts can often make more of an impression that factual news reporting.

Polygenic risk scores (PRS) are a method for using the information in DNA to predict future health outcomes (for an overview of their potential clinical uses, see Lambert et al. 2019, and for a discussion about ethics and responsible use, see Adeyemo et al. 2021). PRS exist for a range of diseases (e.g., cardiovascular disease; Sun et al. 2021), as well as non-disease traits (e.g., musical ability; Niarchou et al. 2022). The PRS are created by sequencing (determining the exact sequence of bases in a DNA molecule) or genotyping (determining which common genetic variants an individual carries) DNA from samples donated by many individuals together with information collected about their phenotypes (observable traits). In order to apply the PRS for individual predictive purposes, the patient would also need to obtain their genomic data. Consent for sequencing/genotyping is needed both in the research to create new PRS and in clinical practice to apply an existing PRS diagnostically to a given patient.

The use of PRS in the UK is increasing; for example, via home DNA testing kits, where a consumer pays to receive information about their ancestry, disease risk and other traits. Genetic data may also become routinely collected in studies such as Genomics England’s pilot programme for screening all newborn babies through genetic sequencing (Genomics England 2021) or the HEART study piloting the use of PRS as part of the National Health Service (NHS) health checks (Fuat et al. 2022). The UK Government has produced a document exploring the potential uses for PRS from insurance to sport to education (Government Office for Science 2022), and the UK’s new healthcare strategy has tasked the NHS with incorporating genetic sequencing into routine clinical care (Alderwick and Dixon 2019). However, despite the enthusiasm and potential utility of many PRS, they are not yet ready for implementation in the clinic (Moorthie et al. 2019). One reason for this is the challenge to healthcare professionals to confidently and competently communicate genetic risk to colleagues and patients (Bridgen et al. 2021). A recent paper on the responsible use of PRS highlighted the importance of risk communication to avoid patients suffering physical, financial or psychosocial harm (Adeyemo et al. 2021).

Inspired by the work “Research Usually Sits on Shelves, Through the Play It Was Shared. Co-producing Knowledge Through Post-show Discussions of Research-Based Theatre” (Hundt et al. 2019), we examined theatrical and fictional approaches to encourage bi-directional dialogue about PRS within a PPIE framework and promote the exchange of ideas over a one-way knowledge transfer from researchers to a lay audience. We explored, with a youth theatre group and patients/members of the public, the use of two improvised plays in shaping discussions around PRS being used in clinical settings. The plays were supplementary to a more traditional PPIE workshop featuring a PowerPoint presentation. The intention was not to explore theatre as a tool for impacting public health communication through knowledge transfer (as in Stuttaford et al. 2006 and Colantonio et al. 2008), but as a tool within the PPIE framework to enhance communication between researchers and patients/public with the intention of eliciting ideas and change in research priorities and culture.

## Creating the plays

“I haven’t resolved what I would want to know. I haven’t resolved if I would want to know.” Genetic Destinies play monologue.

An improvisational theatre workshop was held in October 2021 with Babolin Theatre’s youth group (ages 16–19), two patient advocates with Sickle Cell Disease (a genetic condition), a researcher (statistician working with genetic data) and a PPIE Lead from the Cardiovascular Epidemiology Unit of the University of Cambridge. There were two sessions. In the morning, there were warm-up exercises for all attendees, followed by a presentation of PRS background material. In separate rooms, the researcher and the PPIE Lead were asked questions by the theatre students in a press-conference style interrogation (focusing on positive vs negative arguments). This was used by the theatre students to write a draft of the piece “Genetic Destinies”, while the patients, researcher and PPIE Lead discussed questions and ideas that had been raised during the morning session. In the afternoon, there were further warm-up exercises and a discussion of how PRS information is presented to patients, using the example of coronary artery disease risk. This discussion was used by the theatre students to create a second piece (“A Good Life”). The two pieces were performed for a small audience and recorded.

The two pieces, “Genetic Destinies” and “A Good Life”, can be viewed here: www.youtube.com/watch?v=HD3sEo6iY48 (starting at 57:28).

“Genetic Destinies” is a 4-minute monologue. The actor stands at a podium and attempts to discuss PRS, but is quickly lost in his own thoughts and worries. The piece was created out of responses from the researcher/PPIE Lead during a press-conference style interrogation by the theatre group and remixed by the actor into a more abstract portrayal of someone so paralysed by the potential benefits and harms that they are unable to communicate clearly.

“A Good Life” is a 15-minute reactive piece between six actors. It was created through a group discussion between all participants of how PRS information is presented to patients, using the example of coronary artery disease risk. This was followed by each student writing down their thoughts about the discussion and then meeting as a group to create the piece. In “A Good Life”, each actor has a story of the life of “A”, who discovers their PRS for cardiovascular disease and chooses to change, or not change, their lifestyle. The actors interrupt each other, disagreeing on what are the key components of well-being—healthy living, hedonism or more time spent with family. As each version of “A” dies, their narrator leaves the stage declaring “that was a good life”.

## Using the plays in the PPIE workshop

The theatre workshop researcher and PPIE Lead, and an additional researcher (a health data scientist specialising in polygenic scores), facilitated an online PPIE workshop with 25 patients/members of the public in March 2022 to reflect on attitudes towards the potential uses and utility of PRS using the theatre pieces as a communication tool. Several pages of pre-reading were sent in advance. During the workshop, a short PowerPoint presentation on the ideas in the pre-reading was delivered, and participants were shown videos of the two theatre pieces. Verbal feedback was gathered in breakout groups and 16 participants returned written evaluation forms after the workshop (Supplementary C). Further details of the composition of the groups and details of the workshop can be found in Supplementary A.

## Use of theatre to communicate risks

“Overall, I thought about [PRS] from more angles, which made me less sure about how to tackle it ethically. As I had read the material and had the talk before the plays, [the plays] didn’t help my understanding as such, but they were wonderful at making me think!” Public participant written feedback following the PPIE workshop.

PPIE workshop members were strongly in favour of the use of theatre to explain PRS. Some felt the theatre pieces were sufficient in themselves, while others at the PPIE workshop felt that the pieces needed to be broadened and further explained to connect to a wider audience. There was a clear contrast in the quantitative feedback; the pre-reading and PowerPoint presentation were judged more informative (Supplementary D: graph 1), but the theatre pieces created larger shifts in whether public participants felt positively or negatively about PRS (Supplementary D: graph 2). While the former could be explained by the order of availability to the public participants, this would not account for the subsequent emotional changes in perceptions of these issues. This could also be driven by how the researchers chose different questions to start the discussions when using fictional narratives. Understanding about how the pre-reading, presentation and theatre pieces impacted the public participants’ emotional reactions to PRS is important, as their reactions may influence their willingness to donate samples or participate in testing opportunities. The knowledge could also help governments, health agencies or researchers develop communications for asking the public to participate in these activities. It is unclear whether the theatrical performances caused a change in emotions by illuminating aspects about PRS that would have gone otherwise unconsidered or simply sped up their emotional conclusions. In either case, there is a value in researchers, PPIE Leads and patients/members of the public taking to the time to explore PRS in creative ways, as it allows everyone to introspect, process and discuss as a group.

The researchers found the experience positive. In previous PPIE workshops, one researcher felt under pressure to present only as an expert and talk only about facts she knew to be evidenced in research. In contrast, talking about the plays with the public participants allowed her to relax, and she found the experience “liberating”. She noted that there was more freedom to talk openly about the potential negative impact of her work and of misinterpretations of her work, even where those fears might be unrealistic. This framing of the topic supplied the researchers with more targeted feedback in this area than neutral questions might have gained. Freeing academics to speculate and articulate worries is an interesting potential benefit of fictional narratives, as argued by Staley et al. 2017 that the impact of PPI on the behaviours and emotions of researchers may be of greater importance than its impact on a specific piece of research. In Locock et al. (2017), researchers noted the pressure to suppress their opinions in discussions with patients/members of the public and the perception by patients/public of researchers as robotic/unfeeling and needing PPIE activities to humanise their research. While the issue of hierarchy within PPIE is known to sometimes silence public participants (Locock et al. 2017), it is possible that it also silences those with expertise, to the detriment of the overall exchange of ideas.

Previous workshops facilitated by the PPIE Lead, covering a variety of health and data topics, almost always exclusively used PowerPoint presentations. This project was a useful exploration of more creative ways to encourage discussion between all participants. The positive responses to the theatrical plays, and the discussions that they generated, highlighted the value-for-money in spending time and public funds creating less traditional materials, as well as the learning potential for researchers and patients/members of the public. Similar to the researcher, the PPIE Lead also noted that this format empowered her to contribute her own thoughts and feelings during the discussions, instead of participating as an external observer. This insight will be useful in creating future materials and workshops.

Theatre group members (ages 16–19) were overwhelmingly positive about learning complex genetic ideas through theatrical devising, describing it as “intense”, “fun” and “interesting to learn about”. Prior to the theatre workshop, they had identified genetic concepts (e.g., heritability, familial and individual risk) as something they would learn about from the news or science lessons, but did not expect to understand. The theatre group director had also been concerned that the strong technical nature would make it difficult to create a narrative. However, feedback from the actors showed that they were surprisingly pleased with how interesting they found the activity, with multiple references that it made them think more about the real-world ethical implications.

The review of the discussions and subsequent feedback strongly suggested that theatrical and creative storytelling added value to the experience for both the theatre group members and PPIE workshop members, just through creating and watching the short videos. The plays deepened the dialogue between the researchers and the other participants and enabled all parties to better understand our shared concerns about PRS.

Feedback about the theatre pieces and PPIE workshop also identified topics that were underexplored. PPIE workshop members felt more exploration was needed into how one’s relationship to risk changes over a lifetime, particularly around issues like having children (a common reason people seek out genetic testing in the UK). Several also said that understanding the plays would be difficult without some familiarity of the underlying concepts. There were also multiple recommendations to include a glossary of terms found in the pre-reading and that the pre-reading should include bespoke images or a short video explanation.

We identified three key questions about concerns around PRS from both the theatre group and PPIE workshops. Aspects of these questions arose separately in the workshops, despite the different activities (creating the plays versus watching the plays).

- “Can the data be trusted?” included concerns around data security, accuracy and diversity. Negative emotions like anxiety and panic were associated with the storage of genetic data and how private companies might misuse it, while general practitioners (GPs) were seen as “safe holders” of knowledge. Accuracy was discussed in a very binary fashion; that data should be very accurate simply because it is DNA and that if there was not a high accuracy in results then PRS should not be used at all. A second aspect of accuracy arose around ethnicity and how a lack of diversity in research data might increase inequalities in health outcomes. This was a particular concern for the Black patients who joined the theatre group.

- “Does knowing genetic risk actually help the patient?” debated if individuals would want to know their risk score and the potential impact on friends and family. Some did want to know PRS information, while others felt it would not be helpful unless there was new definitive action that could be taken beyond the known advice to make healthy choices. Stronger evidence of good outcomes in practice was wanted. The younger theatre group members more often discussed this as the impact on their own life, while the older PPIE group members discussed the potential fallout on family members.

- “What makes a life worthwhile?” explored how individuals evaluated what was a good life and the cost-to-benefit of PRS versus using the funds to benefit public health in more immediate ways. There was a clear desire for a holistic approach to the usefulness of PRS in terms of life improvement, not just with regard to a single health outcome or health outcomes in general. Both groups expressed that learning their PRS may not change decisions already made about future health benefits versus denial of current pleasures, especially if health advice (eat well, exercise, etc) remained the same.

Detailed discussions about these key questions can be found in Supplementary B.

## Conclusion

“Maybe if they had exercised more or quit smoking completely, they would have lived for longer. But what would have been the point?” A Good Life play.

The two plays developed with the theatre group were quite simplistic. However, creating and viewing fictional narratives helped both the theatre group and PPIE workshop members explore their own feelings about the information rather than focusing on improving how much they learnt. The variety of responses induced by the plays and the large well of uncertainty and negative responses from the PPIE workshop members will help guide future PPIE activities, as well as PRS research, particularly in the rush to push PRS into clinical practice. Future workshops will explore the hesitancy and distrust of PRS and will use this knowledge to inform design and communication around implementing PRS research.

Further work on the plays is dependent on future funding, but collaborations using fiction and/or art to help the public explore their thoughts and concerns about PRS could potentially have a large value as these new health tools become more frequently and widely used. Creating narratives in collaboration with under-served groups should be prioritised to reduce the information and health inequalities in their communities (Kaplan et al. 2017), such as our own deliberate inclusion of chronically ill and Black collaborators in this project. There are several existing, well-produced videos about PRS for lay audiences on YouTube (British Heart Foundation 2017; Illumina 2019; Harvard Medical School 2020; CPM Oxford 2021), but all are factual pieces. We aim to employ a combination of fiction and non-fiction videos in future PPIE activities to help people become familiar with the material.

Theatre is just one approach to art-based communication in research; collage (Dutton et al. 2019), sewing (Brubacher et al. 2021), poetry (Faulkner 2017) and other art forms may also have similar utility in encouraging and supporting dialogue within PPIE activities. We encourage other research teams to create a wider array of materials for discussing their work and to consider the utility of realistic fictional narratives and theatrical works for facilitating bi-directional dialogue with lay audiences.

### Supplementary information

ESM 1 (DOCX 55 kb)
