# Supplementary material for: What makes a good life: using theatrical performance to enhance communication about polygenic risk scores research in patient and public involvement
Source: J Community Genet. 2023 Feb 10;14(5):453–8. doi: 10.1007/s12687-023-00635-1 (PMC10576689; doi:10.1007/s12687-023-00635-1)
Supplement: Supplementary file 1 — (DOCX 55 kb) [file 12687_2023_635_MOESM1_ESM.docx]

**Supplementary A: Description of play creation and use in PPIE**

**Improvisational theatre workshop**

This workshop was run as part of Cambridge Creative Encounters – Behind the Curtains ([www.cam.ac.uk/public-engagement/creative-encounters-2022/behind-the-curtains](http://www.cam.ac.uk/public-engagement/creative-encounters-2022/behind-the-curtains)), a collaborative partnership with Babolin Theatre ([www.babolintheatre.com](http://www.babolintheatre.com)) to produce theatre pieces reflecting research at the University of Cambridge. Babblers, part of Babolin, is an improv group run for Hills Road Sixth Form College ([www.hillsroad.ac.uk](http://www.hillsroad.ac.uk)) students. The theatre group (ages 16-19) worked with two individuals with Sickle Cell Disease, a researcher (statistician working with genetic data) and a PPIE Lead. Cambridge Creative Encounters provided a unique opportunity for the researcher and PPIE Lead to work with young people, in contrast to their groups of older members of the public, yielding different perspectives about the use of data and disease risk. The two individuals with Sickle Cell Disease were selected from a group of regularly transfused patients involved in research, to represent people managing a genetic disorder and often unheard perspectives. One individual attended via an online platform (Zoom) and one attended in-person. Both were sent information about the project before working with the theatre group.

The theatre workshop involved two sessions. In the morning, there were warm-up exercises for all attendees, followed by a presentation of PRS background material. In separate rooms, the researcher and the PPIE Lead were asked questions by the theatre students in a press-conference style interrogation (focusing on positive vs negative arguments). This was used by the theatre students to write a draft of the piece “Genetic Destinies”, while the patients, researcher and PPIE Lead discussed questions and ideas that had been raised during the morning session. In the afternoon, there were further warm-up exercises and a discussion of how PRS information is presented to patients, using the example of Coronary Artery Disease risk. This discussion was used by the theatre students to create a second piece (“A Good Life”). After practice performances of both pieces, everyone contributed verbal feedback and evaluation. The theatre group members evaluated the experience by using art supplies to create posters and the patients provided written feedback after the workshop (Supplementary C). A final performance of the pieces was recorded several days later.

**Online PPIE workshop**

Email invitations were sent to groups of patients/members of the public who were already involved in research due to the technical and unfamiliar nature of PRS. Participants were offered an honorarium of £50 per person involvement fee, £5 remote working costs and offered other payment (childcare, carer costs, etc) to support inclusivity, as per the National Institute for Health and Care Research’s Payment Guidance (National Institute for Health and Care Research 2022).

One week before the workshop the 25 participants were sent details about the workshop structure and background information on PRS, communication of risk and the research currently being conducted at the University of Cambridge (available upon request). This was the same material as had been used in the theatre workshop to prompt the creation of the pieces. The workshop was hosted online using a remote platform (Zoom) and lasted approx. two hours. An introduction outlined the aims and structure of the workshop, a short PowerPoint presentation repeated the background information about PRS that had been sent before the workshop, there were discussions in breakout rooms and as a whole group, the participants were shown the theatre pieces (“Genetic Destinies” and “A Good Life”) and there was a final breakout room and discussion as a whole group.

After the PowerPoint presentation, participants were randomly split into three breakout rooms and asked to focus on a difference topic:

1. How do you feel about PRS? Frightened? Hopeful? Confused?
2. Would you want to know your PRS for a specific disease? How would it change your behaviour of choices?
3. How would you describe a PRS to a family member or friend? Would you feel confident discussing a PRS with your GP?

After viewing the theatre pieces, they were asked to discuss:

1. Which piece did you prefer? Why?
2. What was the meaning of each piece? Did they make you feel or think differently about PRS?
3. How might finding out our PRS change us? When should we use PRS in our lives?

Following each breakout room discussion, each room fed back to the main group and there was an opportunity to ask the researchers questions. The breakout rooms were not recorded, in order to encourage dialogue, but notes were taken by the researchers and PPIE Lead with the consent of the participants. No personal or identifiable data was collected with the verbal notes.

Following the workshop, participants were asked to return written evaluation forms with feedback about the handouts, presentation and theatre pieces and demographic information. The feedback forms were anonymised before shared with the researchers and demographic information was securely stored and only available to the PPIE Lead. The demographic information helped the PPIE Lead understand who participated in the PPIE workshop and will help improve future communications and public activities. It also highlighted groups that were under-represented (National Institute for Health and Care Research 2020). A copy of the feedback form is in Supplementary C. Participants consented to anonymised information being shared.

25 patients/members of the public participated in the online PPIE workshop. 16 returned evaluation forms. 5 men and 11 women. 12 people were over 50 and 4 were between 20-50. The majority were white (14), 50% had children (8) and 6 were either disabled or had a genetic condition. Most were not health professionals (13).

Key questions about concerns around PRS were identified from the verbal feedback from the theatre group, discussions during the online PPIE workshop and written evaluation forms. Following analysis, summaries of the identified questions were sent to all participants (Supplementary B).

National Institute for Health and Care Research (2020) Improving inclusion of under-served groups in clinical research: guidance from INCLUDE project. https://www.nihr.ac.uk/documents/improving-inclusion-of-under-served-groups-in-clinical-research-guidance-from-include-project/25435. Accessed 13 June 2022.

National Institute for Health and Care Research (2022) Payment guidance for researchers and professionals. https://www.nihr.ac.uk/documents/payment-guidance-for-researchers-and-professionals/27392. Accessed 13 June 2022.

**Supplementary B: Key questions summary**

**1. Can the data be trusted?**

*“Dystopia, liberation, paranoia, knowledge. Safety, data and how it is used.”* Genetic Destinies play monologue.

A. Data security

Worries about the secure storage and appropriate use of data was mentioned by one of the theatre group members and several public participants during the online PPIE workshop. The theatre group member compared their worries about the massive volume of data generated to create PRS to Google’s use of internet history data to make unexpected predictions. The public participants expressed negative emotions such as anxiety, panic and fear, as well as worries about private companies accessing and misusing data and specific fears like losing jobs or struggling to get insurance due to widespread availability of their personal data.

This contrasted heavily with public participants’ attitudes to GPs having the data. Many mentioned that they would prefer their GPs only to know the results while they remain ignorant of their PRS (see “Who, what and when” below).

Two public participants brought up a different aspect of data security – how to ensure that data isn’t tampered with or in error. One linked their concern to celebrity and tabloids, suggesting that data could be fabricated and shared to papers, while the other mentioned laboratory error (linking it to the accuracy point below). The concern about laboratory error is a striking one; for cost-efficiency purposes, there is a good reason to sample people once and then use that data throughout their lives to create PRS for disease outcomes. However, an error at this stage could persist for decades – as it would be difficult for either patients or primary care physicians to identify – and could serially impact on one’s understanding of their health.

*“My feelings and ideas have changed on the pros of Polygenic Risk Scores. I think the pros are more powerful, but the cons are a lot in the wrong companies and hands.”* Public participant written feedback.

B. Accuracy

There was an interesting split in opinions by the public participants around concepts of accuracy. Some felt that because PRS were DNA-based, they should be more accurate than other measures. Others thought that the technology couldn’t be trusted yet and that it wouldn’t give better information than a family history. This was often linked with worries that poor communication by health professionals around accuracy and risk could lead to under, or over, trust in the results by patients.

There was often an element of black/white thinking here, either that if people were told a PRS by a health professional it would be assumed to be completely accurate, but that any lack of accuracy makes the PRS unsuitable and it shouldn’t be used at all. The nuance of uncertainty in medical interpretation of results was something that multiple public participants were concerned with, and they worried about how much patients and health professionals would be able to understand. This also appears in the theatre piece “Genetic Destinies” – “We shouldn’t disregard a tool just because it isn’t perfect”.

C. Diversity of the data

Multiple public participants mentioned the lack of diversity in the data, resulting in less accurate PRS for people of non-white ancestry. Concerns centred both on the way this could increase health inequality and impact confidence in the use of PRS. Both of the patients working with the theatre group both felt more negative about PRS after their experience, referencing the lack of ethnic diversity in the data and the effect of that on accuracy.

*“…it seems positively dangerous to use PRS at the moment unless they are based on data obtained from people with the same ethnic background mix.”* Public participant written feedback.

**2. Does knowing genetic risk actually help the patient?**

*“I haven’t resolved what I would want to know. I haven’t resolved if I would want to know.”* Genetic Destinies play monologue.

A. Who, what and when

A repeated theme in the online PPIE workshop was around what PRS they would want to know. There was a distinction between illnesses where there was useful action that could be taken, and a clear fear that knowing about diseases that couldn’t be prevented would lead to stress and distress. Interestingly, this fear is contradicted by current research (Frieser et al. 2018), which found little evidence of negative behavioural changes or psychological impact. The fear of knowledge of high genetic risk of disease may be similar to the fear of diagnosis of uncurable disability in general, which can cause poor mental health outcomes in the short-term, but can be recovered with acceptance of disability and disease (Koziel et al. 2016). This second point can be linked to comments by public participants about the “worried well” and companies preying on health fears, as well as the privatisation of health services in the UK.

There were multiple suggestions that GPs could be instructed to not pass on PRS to patients unless a specific threshold of risk was reached, or that PRS should be used to plan screening frequency, but should not be known by the GP or patient. One public participant raised concerns over GPs being sued if they knew something and didn’t tell the patient. A lot of the value of PRS was placed on points where there is clear action; for example, changing screening opportunities or treatment options.

Several public participants questioned the value of knowing their PRS even in cases where there was action to be taken, since GPs have been giving the same general advice for decades (stop smoking, stop drinking, eat healthier, do more exercise). Others disagreed and said they would take deliberate action if told that they were at higher risk of developing a disease. One male public participant used the example that he would start breast self-exams if he knew he was at high risk of breast cancer but wouldn’t otherwise. One patient, who worked with the theatre group, shared that they often talk to their children about diet, exercise and being mindful of who to have children with because of their family history of genetic disease. Interestingly, a disabled public participant said that their health issues made them less inclined to find out about genetic risks, as this is already a big part of their life.

Discussions around whether or not public participants would want to know their PRS led to a discussion about the cost/benefit of generating PRS. Public participants wanted evidence that PRS would lead to improved outcomes and not just improved knowledge. Widén et al. (2022) showed that knowledge of cardiovascular disease PRS led to improved health choices, although it has not been shown whether these will be maintained long enough to improve outcomes. CanRisk is currently running a trial in Canada to see if knowing genetic cancer risk (as part of a larger PRS) leads to better cancer outcomes (Archer et al. 2020).

*“Getting a Polygenic Risk Score means you either let it take over your life or not or live in the moment.”* Public participant during online PPIE workshop.

B. Impact on friends and family

While theatre group members, who were aged 16-19, primarily related the potential impact of PRS to their own lives, the public participants, many of whom were >50 years old, brought up the fallout on other family members. Many felt that it would be harder to know this information about someone else than about themselves. There was a repetition of some of the earlier mentioned fears around having knowledge, but not having the agency to change anything. One public participant tied this back to what GPs should tell patients and the impact of a patient knowing something that could affect other family members. There were also questions over the ethics of a GP not telling a patient something if the information was gained through the PRS of a family member. The confidentially of a doctor is not absolute; for example, it is considered ethical for a GP to inform a patient that their partner has HIV without the partner’s consent. The issues around this will need to be resolved by GPs, particularly around to what extent the probabilistic nature of PRS affect the balance of confidentially versus risk of harm to other patients.

**3. What makes a worthwhile life?**

*“If you want to live in [a] bubble to get [an] extra 5 years, you can. If you want to drink and smoke and go out with [a] bang, you can!”* Public participant during online PPIE workshop.

A. Healthspan/lifespan

The question of what makes a worthwhile life was a main focus for theatre group members, who acted out various scenarios in their theatre piece “A Good Life”, and it was echoed both during the online PPIE workshop and in the written feedback forms. This theme drove much of the positive feedback from the public participants about the theatre pieces – “add life to our years, not necessarily years to our life” and “healthspan/lifespan”. This may represent a desire for different sorts of outcomes to be presented as part of health analyses; for example, not just years of life, or even years of disability-free life, but some way of judging whether the adaptions needed to improve health were worth what you are giving up.

There was duality of thought around what the public participants thought and what the public participants expected other people to think about very high or very low PRS. They commented more on how they thought people in general would behave rather than reflecting on their own likely behaviour. Some public participants felt those with low PRS might increase their “wild lifestyle” and thus end up compromising their innate better health, while others felt that knowing you were likely to die early might influence people to “live life to the fullest”. This echoes the theatre group’s piece “A Good Life”, where characters express differing responses to high PRS with some giving up smoking or moving to lower stress jobs, and others refusing to believe it or quickly abandoning unrealistically strict^[[1]](#footnote-2)^ diets for junk food. The greatest benefit to PRS that theatre group members and public participants articulated was around the opportunity for choice, that greater knowledge might lead to better outcomes not in terms of health, but in terms of a lack of regrets in the life lived.

B. Cost to benefit

One of the public participants brought up the high cost of genetic sequencing and suggested that people should be surveyed to determine if they would rather that the £750 was spent on getting their genome sequenced or put towards their food and heating bills. This is a difficult public health question, especially at a time when costs of living are rising and poverty is the single largest impact on health (World Health Organization 2008).

On the other hand, points were raised in the online PPIE workshop about the risks of sequencing becoming the sole territory of private health research companies and the affluent being the sole beneficiaries of the principal ‘benefit’. Linked to the context of accelerating privatisation and outsourcing of parts of the NHS, there was concern over increasing health inequalities.

Archer S, Babb de Villiers C, Scheibl F, Carver T, Hartley S, Lee A, et al (2020) Evaluating clinician acceptability of the prototype CanRisk tool for predicting risk of breast and ovarian cancer: A multi-methods study. PLoS One 15(3):e0229999. https://doi.org/10.1371/journal.pone.0229999

Frieser MJ, Wilson S, Vrieze S (2018) Behavioral impact of return of genetic test results for complex disease: Systematic review and meta-analysis. Health Psychol 37(12):1134-44. https://doi.org/10.1037/hea0000683

Koziel P, Lomper K, Uchmanowicz B, Polanski J (2016) Association between acceptance of illness, anxiety and depression with assessment quality of life of breast cancer patients. Medycyna Paliatywna w Praktyce 10(1):28-36.

Widén E, Junna N, Ruotsalainen S, Surakka I, Mars N, Ripatti P, et al (2022) How Communicating Polygenic and Clinical Risk for Atherosclerotic Cardiovascular Disease Impacts Health Behavior: an Observational Follow-up Study. Circ Genom Precis Med 15(2):e003459. <https://doi.org/10.1161/CIRCGEN.121.003459>

World Health Organization (2008) Closing the gap in a generation: health equity through action on the social determinants of health – final report of the commission on social determinants of health. https://www.who.int/publications/i/item/WHO-IER-CSDH-08.1. Accessed 13 June 2022

**Supplementary C: Written evaluation form**

**Pre-reading**

1a. Did this raise your **awareness and understanding** of Polygenic Risk Scores (PRS)?

Very well  Well  Not very well  Not at all  I didn’t read it

1b. Did this change **how you feel** about PRS?

Much more positive  A bit more positive  No change

A bit more negative  Much more negative

**Zoom presentation**

2a. Did this raise your **awareness and understanding** of PRS?

Very well  Well  Not very well  Not at all

2b. Did this change **how you feel** about PRS?

Much more positive  A bit more positive  No change

A bit more negative  Much more negative

**Plays**

3a. Did they raise your **awareness and understanding** of PRS?

Very well  Well  Not very well  Not at all

3b. Did they change **how you feel** about PRS?

Much more positive  A bit more positive  No change

A bit more negative  Much more negative

4. We are really interested in the best way to **communicate** PRS. What idea, analogy or method used today in the workshop helped you understand? Do you have any ideas on how to explain it better?

5. Do you have **any other comments** you would like to share?

6. Would you be interested in attending **a future workshop** about ethical questions relating to PRS?

Yes  No

**Supplementary D: Feedback graphs**

Graph 1. Results from written feedback questions 1a, 2a and 3a on how the pre-reading, presentation or play impacted their (patients/public) *understanding and awareness* of PRS. Counts of number of respondents shown under each section.

Graph 2. Results from written feedback questions 1b, 2b and 3b on how the pre-reading, presentation or play changed how they (patients/public) *felt* about PRS. Counts of number of respondents shown under each section.

1. “”A” ate only yogurt. Their parents gave them only yogurts. All establishments had to give them yogurt”. [↑](#footnote-ref-2)
